# Metformin in Reproductive Biology

**DOI:** 10.3389/fendo.2018.00675

**Published:** 2018-11-22

**Authors:** Melanie Faure, Michael J. Bertoldo, Rita Khoueiry, Alice Bongrani, François Brion, Cecilia Giulivi, Joelle Dupont, Pascal Froment

**Affiliations:** ^1^Unité de Physiologie de la Reproduction et des Comportements, Centre Val de Loire, Institut National de la Recherche Agronomique, UMR85, Nouzilly, France; ^2^Discipline of Obstetrics and Gynaecology, School of Women's and Children's Health, University of New South Wales, Sydney, NSW, Australia; ^3^Department of Development and Regeneration, Stem Cell Institute, KU Leuven, Leuven, Belgium; ^4^INERIS, Direction des Risques Chroniques, Pole VIVA, Unite d'ecotoxicologie in vitro et in vivo, BP2, Verneuil-en-Halatte, France; ^5^Department of Molecular Biosciences, School of Veterinary Medicine, University of California, Davis, Davis, CA, United States; ^6^Medical Investigations of Neurodevelopmental Disorders Institute, University of California, Davis, Davis, CA, United States

**Keywords:** testis, ovary, metformin, oocytes, spermatogenesis

## Abstract

Initially produced in Europe in 1958, metformin is still one of the most widely prescribed drugs to treat type II diabetes and other comorbidities associated with insulin resistance. Metformin has been shown to improve fertility outcomes in females with insulin resistance associated with polycystic ovary syndrome (PCOS) and in obese males with reduced fertility. Metformin treatment reinstates menstrual cyclicity, decreases the incidence of cesareans, and limits the number of premature births. Notably, metformin reduces steroid levels in conditions associated with hyperandrogenism (e.g., PCOS and precocious puberty) in females and improves fertility of adult men with metabolic syndrome through increased testosterone production. While the therapeutical use of metformin is considered to be safe, in the last 10 years some epidemiological studies have described phenotypic differences after prenatal exposure to metformin. The goals of this review are to briefly summarize the current knowledge on metformin focusing on its effects on the female and male reproductive organs, safety concerns, including the potential for modulating fetal imprinting via epigenetics.

## Introduction

### Brief history of metformin

The insulin-response sensitizer metformin (*N*,*N*-dimethylbiguanide) has been an important drug for the treatment of diabetes since the 1950's, being one of the most widely prescribed anti-hyperglycemic compounds. Metformin belongs to the biguanide family of anti-diabetic compounds that are related to galegine, a guanidine derivative from the French lilac (*Galga officinalis*). In the beginning of the twentieth century, a chemical study of active molecules contained in *Galga officinalis* demonstrated anti-hyperglycemic properties in diabetic patients (1). Metformin synthesized in 1958, showed similarities with galegine and lowered blood glucose in initial tests on animals (2, 3). Metformin decreases the glycemia through a reduction in hepatic gluconeogenesis and intestinal glucose absorption, with a general improvement in tissue insulin sensitivity and peripheral glucose uptake (4). It is a stable, low molecular weight hydrophilic compound, which upon administration to patients, it reaches numerous tissues including muscle, liver, pancreas, adipose tissue, hypothalamus, pituitary, and the gonads.

### Cellular targets of metformin

**Figure 1:**
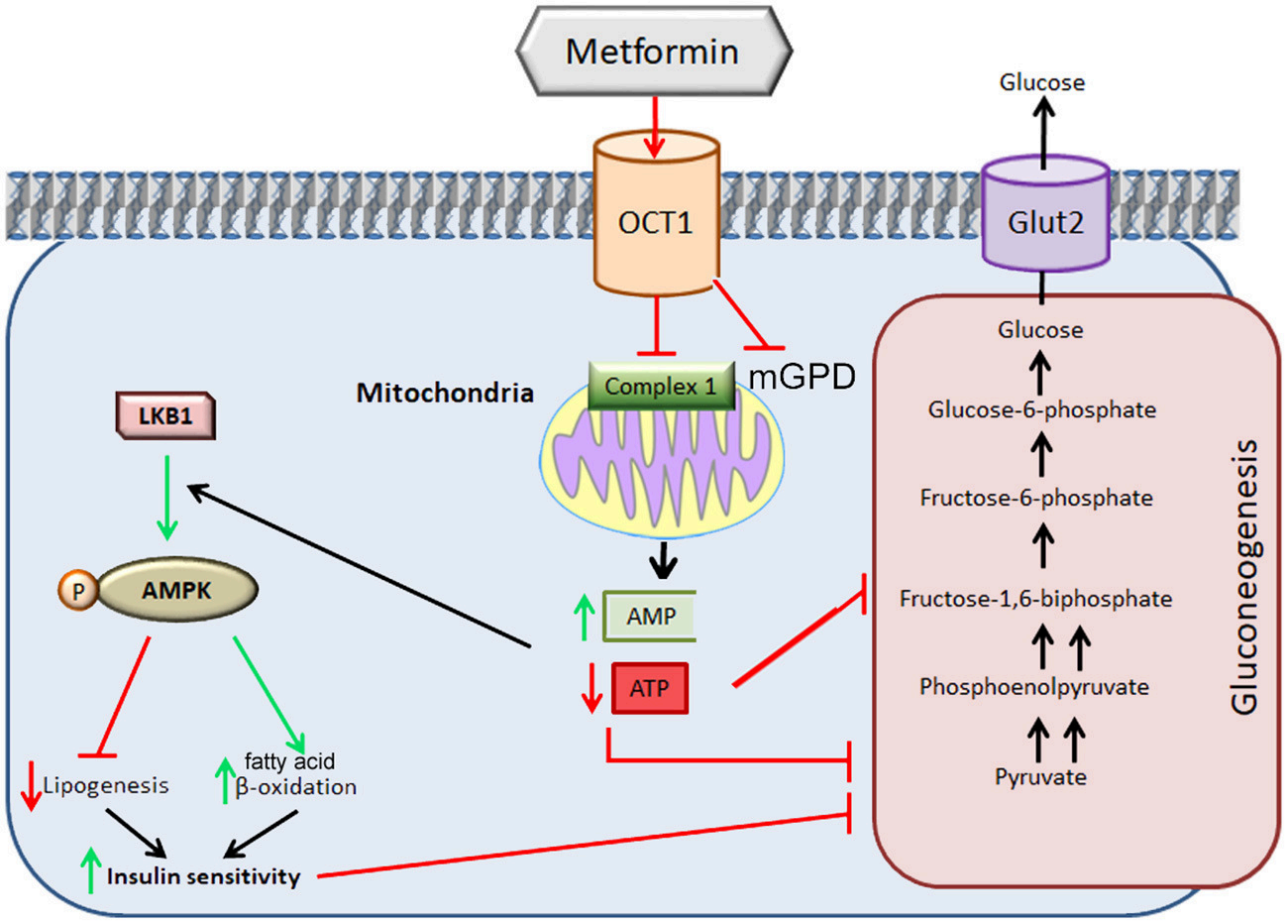
Metformin-induced inhibition of mitochondrial Complex I. The direct inhibition of Complex I by metformin decreases the production of ATP ensuing in increases in AMP. The increase in the [AMP] to [ATP] ratio signals energy resulting in inhibition of high-energy demanding gluconeogenesis process. This ratio leads to the activation of the AMPK complex leading to a decrease in lipogenesis, increase in fatty acid beta-oxidation with an improvement in insulin sensitivity which allows the restoration of gluconeogenesis. The inhibition of metformin on mGDP prevents the use of lactate or glycerol for gluconeogenesis. OCT1: Organic Cation Ttransporter 1*;* LKB1: Liver Kinase B1*;* Glut2: GLUcose Transporter 2. mGPD: mitochondrial glycerophosphate dehydrogenase. Adapted from (20).

The exact molecular mechanism of metformin's action remains unclear. In the first publications, metformin's actions at the cellular level have been attributed to inhibition of Complex I of the mitochondrial respiratory chain, albeit at relatively high concentrations (mM) (5–10). This inhibition results in a decline in ATP production by mitochondria and an increase in the [adenosine monophosphate to ATP ratio ([AMP]/[ATP]) leading to the activation of the AMP-activated protein kinase (AMPK) complex (11). AMPK is a critical cellular energy sensor that maintains cellular energy homeostasis. Following its activation, AMPK initiates energy-producing catabolic pathways including facilitation of cellular glucose uptake and stimulation of glucose transporter expression, glycolysis, fatty acid beta-oxidation, oxidative phosphorylation and mitochondrial biogenesis. This compensatory mechanism aims at restoring sufficient energy to maintain cellular homeostasis (12–15) Figure 1. AMPK activity often counteracts the actions of the mammalian target of rapamycin (mTOR), a central cell-growth factor controlled by extracellular growth triggers and nutrients.

**Figure 2:**
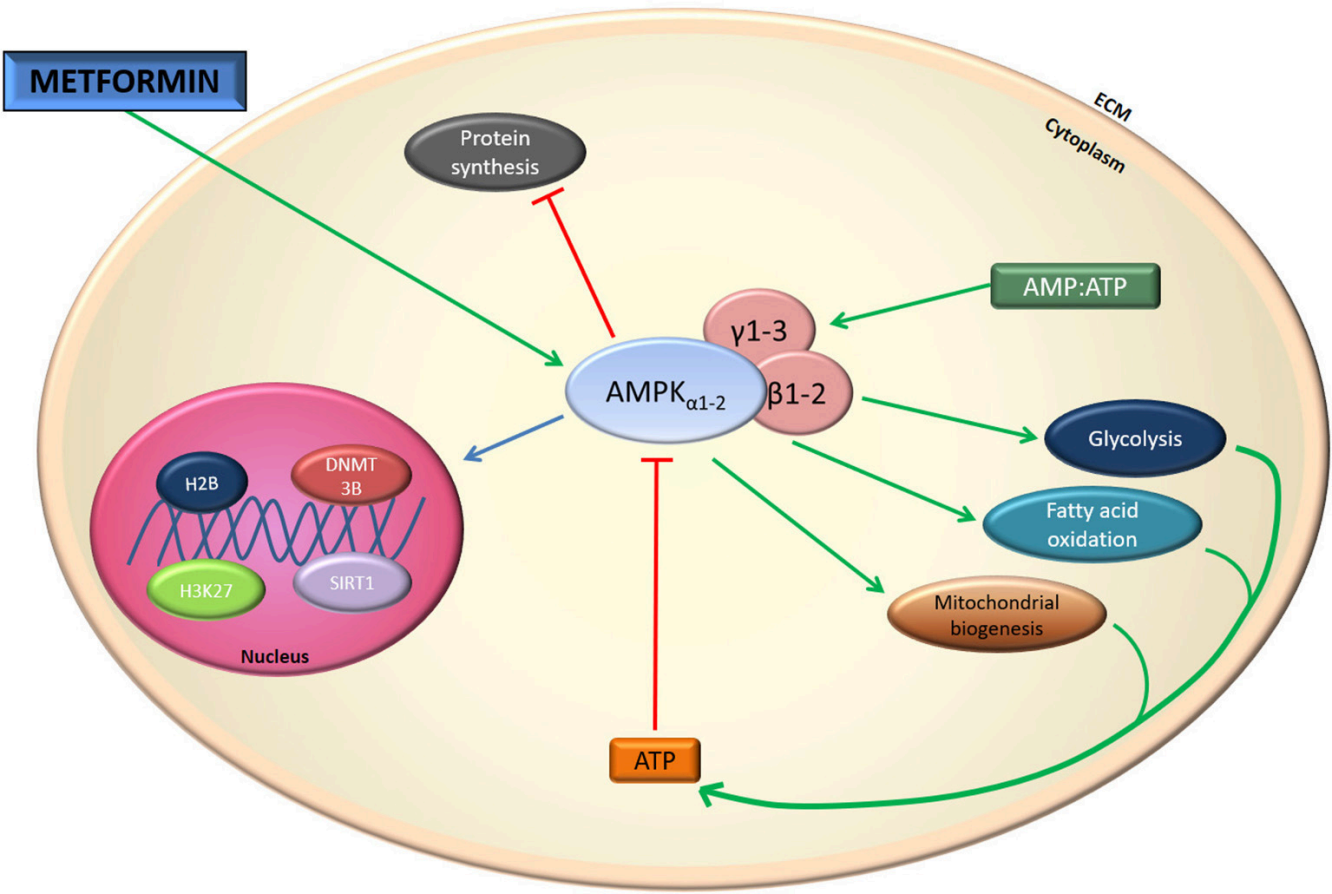
Metformin effect on AMPK. Indirectly metformin activates AMPK. This activation results in mitochondrial biogenesis and glycolysis.

However, the metformin-dependent mitochondrial Complex I inhibition can not account for all of metformin's effects suggesting that metformin may act in an AMPK-independent manner (12–14, 16–20). For instance, it has been described that metformin inhibits the mitochondrial redox shuttle glycerophosphate dehydrogenase. The limited conversion of lactate and glycerol to glucose results in lower hepatic gluconeogenesis (21, 22) (Figure 2). Other studies identified H3K27me3 demethylase, KDM6A/UTX as a metformin target based on a structure- and ligand-based bioinformatic analysis (23). Some of the antidiabetic effects of metformin seem to be mediated in part to changes in gut microbiota, thereby promoting the growth of short chain fatty acid-producing bacteria (24, 25). Other studies reported the effect of metformin on the mitochndrial permeability transition pore (26) whereas others on the effects of this drug on cell death (27).

## Absorption and distribution of metformin

Metformin is used at daily doses of 30–50 mg/kg body weight to treat type II diabetes, reaching serum levels of 10–40 μM (28, 29). It is absorbed through the small intestine, with peak concentrations 1–2 h after oral administration. Its plasma half-life is about 1–6 h. No metabolites of metformin have been identified, and is excreted as such in the urine within 12 h (29).

Species-specific differences show that mice are ~10 times less sensitive to metformin than humans (20, 29, 30). In mice, after daily administrations at 50 mg/kg body weight, the serum concentrations of metformin are 1.5 and 30 μM for 500 mg/kg (30, 31). In human, 10–40 μM in blood level is reached with 30–50 mg metformin/kg (28, 29). As such, daily administrations of 250–300 mg/kg of metformin to diabetic mice are significantly higher than those used in humans in order to obtain similar therapeutic benefits (30). These species-specific metformin examples are relevant, not only when doses are compared across species but also when interpreting potential effects and targets.

Once in plasma/serum, metformin reaches the intracellular milieu via a limited passive diffusion, while other studies indicated that cationic transporters (Organic Cation Transporter 1: OCT1, OCT2, and MATE1) are able to transport metformin intracellularly (32). It has been claimed that genetic polymorphisms in the genes coding for these transporters may alter the tissue distribution and pharmacological effect of metformin (33).

## Metformin's potential impact on infertility

The bioenergetic, metabolic processes indicated above are critical to sustain a physiological function of the male and female gonads, therefore, in the following sections, we summarize the current knowledge on metformin in regard to its effects on the reproductive processes of males and females in humans and across species. We also discuss the safety of metformin and its potential epigenetic consequences for fetal imprinting.

### Clinical and molecular impact of metformin in the female reproductive system

Since polycystic ovary syndrome (PCOS) is often associated with obesity, metabolic syndrome, gestational diabetes, and T2DM and cardiovascular risk factors, it is not surprising that PCOS patients with insulin resistance and hyperandrogenism are treated with metformin. PCOS is a major health issue affecting ~5–20% of reproductive age women, representing the most common ovarian pathology in the world. Based on the criteria of the 2003 Rotterdam Consensus, PCOS is characterized by at least two of the following three criteria: (a) oligo- or anovulation, (b) clinical and/or biochemical signs of hyperandrogenism, (c) presence of 12 or more follicles in each ovary measuring 2–9 mm in diameter and/or increased ovarian volume (>10 ml) and the exclusion of other etiologies (34, 35). The immediate and short-term effects of metformin in women affected with PCOS are in general beneficial. Metformin's treatment improves ovarian cyclicity and reduces gestational diabetes with no impact on the incidence of cesareans or premature births (36–38). While in some countries, metformin is prescribed during pregnancy to women suffering PCOS and gestational diabetes (39–41), the US Food and Drug Administration (FDA) indicated that the safety of metformin during pregnancy is still unclear (28, 42).

However, metformin's efficacy on ovulation and birth rate alone or in combination with clomiphene citrate, compared to clomiphene citrate treatment alone, is still a matter of debate (43–46). For example, an analysis of nine randomized trials including 816 women with PCOS has shown that metformin increased clinical pregnancy rates and decreased the risk of ovarian hyperstimulation syndrome, although there was no clear beneficial evidence for increased rates of live births (47, 48) or increasing the risk for birth defects (49). The premature birth incidence was higher under metformin therapy (45). A recent meta-analysis described that ovulation rate was significantly higher under a combination of metformin and letrozole treatments than with other treatments (metformin alone, letrozole alone, metformin and clomiphene citrate, FSH, laparoscopic ovarian drilling) in females affected with PCOS resistant to clomiphene citrate treatment (50).

At the cellular level, metformin has been observed to regulate oocyte maturation. During bovine and porcine oocyte maturation, metformin is able to impede meiotic progression (51, 52). In the bovine oocyte, meiotic arrest was associated with an increase in AMPK activity, a reduction in MAPK ERK1/2 phosphorylation in both oocytes and cumulus cells, and the latency of ribosomal protein 6 and EEF2 (Eukaryotic elongation factor 2), two critical factors regulating protein synthesis in oocytes. Moreover, these effects were only evident in cumulus-oocyte complexes and not in oocytes that had the cumulus compartment removed, indicating that at least in the bovine, cumulus cells are key for metformin to access the oocyte (52).

In a mouse model of PCOS, metformin treatment was explored to alleviate the negative influence of hyperandrogenism on oocyte quality (53). Metformin treatment of PCOS-affected dams was also able to partially reverse ovulatory dysfunction and improve oocyte quality and embryo development outcomes (53). These metformin-mediated improvements were associated with a reduction in oocyte lipid content and reactive oxygen species content, and improved mitochondrial function and glutathione levels (53), consistent with the effect of metformin on the oocyte-specific AMPK knockout mouse model (50). While AMPK is expressed in all ovarian compartments across different species (cow, goat, ewe, sow, hen, rat) including women, deletion of the AMPKα1 subunit specifically in oocytes of mice results in a 27% reduction in litter size (54), highlighting the importance of the AMPK complex to oocyte developmental competence and fertility. Moreover, following *in vitro* fertilization of oocyte-specific AMPK KO mice, a 68% reduction in the number of embryos passing the 2-cell stage was observed (54). This decrease in fertility could be partly explained to defective mitochondrial morphology and ATP synthesis (54). These results suggest that metformin could reverse the negative effects of hyperandrogenism on oocytes in PCOS individuals.

Mouse embryos exposed to metformin from the 2-cell to the blastocyst stage *in vitro* are smaller in size with lower cell numbers (55). The cell-to cell contact with trophectoderm was also altered because of an increase in tight junction permeability (55). *In vivo*, metformin reduced apoptosis in blastocysts of obese mice (56) possibly through an increase in NAMPT expression, but induced early bovine embryo arrest (57). Taken together, these studies demonstrate metformin's important contribution in the cross-talk between somatic cells and oocytes for the normal development of high-quality female germ cells and embryo developmental competence.

### Metformin and male reproductive biology

**Figure 3:**
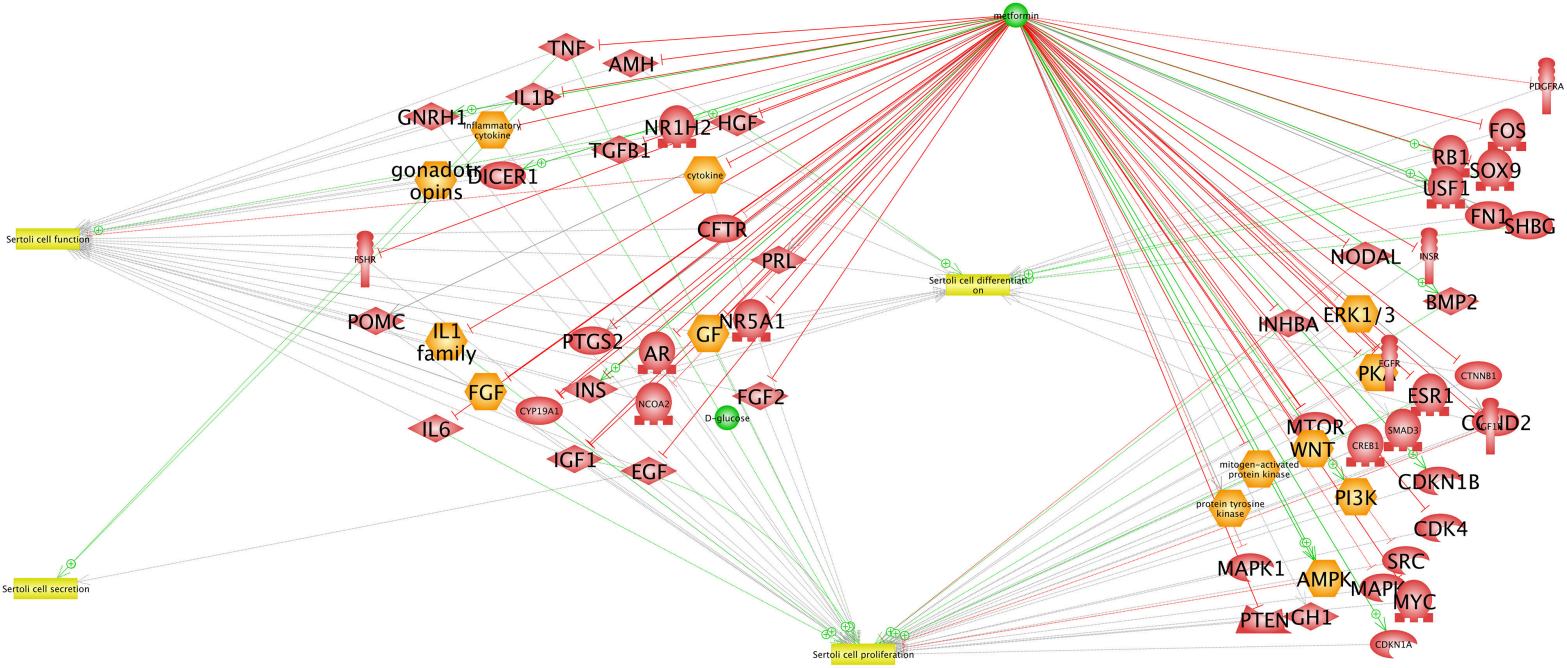
Interaction network between AMPK and proteins expressed in Sertoli cells. The network was created using the Elsevier Pathway Studio program. Green arrows indicate activation while red arrows indicate inhibition.

In males, metformin is prescribed for the treatment of T2DM. It is well-known that T2DM alters spermatogenesis in males, decreasing both sperm number and quality (58–60), resulting in reduced fertility. Furthermore, *in utero* exposure to metformin reduces fetal testicular size and the population of Sertoli cells (SC) (61). It is possible that these processes are driven by metformin-mediated increase in lactate production with a decrease in testosterone secretion (61). Metformin impacts the cell cycle by decreasing FSH-induced proliferation and increasing Cyclin-Dependent Kinase Inhibitor (CDKI) and inhibiting cyclin D in primary cultures of mouse Sertoli cells (62) (see Figure 3).

However, administration of metformin (for 4 or 8 weeks at doses of 100 or 500 mg/kg) to adult, non-obese rats did not impact sperm number, sperm motility, or the percentage of abnormal spermatozoa (63) (see Table 1). In contrast, obesity induced an increase in the number of sperm abnormalities and a decrease in the spermatozoa concentration and motility which was rescued by metformin administration (63). In obese patients, metformin treatment improves sperm concentration and motility in the same way as observed in obese rats (63–66) as judged by the decreased number of morphological defects, with higher concentration and motility of sperm (64). In humans, it appears that a treatment for several months with metformin (850 mg/day during the first week, 1,700 mg/day during the second week, and 2,550 mg/day until the end of 6 months of treatment) can increase the serum testosterone and LH pulsatility of obese individuals (64). This suggests that metformin can modulate and improve pituitary LH pulsatility and regulate Leydig cell steroidogenesis in testis. Recently, it has been shown that metformin decreases sperm motility in pigs (67), correlated with an increase in the viability of the spermatozoa after 24 h storage (see Table 1). In a model of testicular ischemia—stress-triggered apoptosis—metformin pre-treatment reduced both oxidative stress and the loss of germ cells, thereby limiting injury on sperm production, suggesting that metformin has cytoprotective effects (68).

**Table 1:** Effect of *in vivo* metformin treatment on male sperm.

**Species**			**Rat**	**Rat**	**Rat**	**Rat**	**Rat**
Pathology			Diabetic (streptozotocin)	Control	Control	Diabetic (streptozotocin)	Diabetic (streptozotocin)
Metformin treatment			Yes	Yes	Yes	No	Yes
Comparison type			Comparison untreated with metformin	Comparison untreated with metformin	Comparison untreated with metformin	Comparison diabetic with non-diabetic	Comparison untreated with metformin
Administration mode			Gavage 50 mg/kg/day during 4 weeks	Gavage 30 mg/kg/day during 21 days	Gavage 500 mg/kg during 4 or 8 weeks		Gavage 500 mg/kg during 4 or 8 weeks
Sperm	*In vivo*	Morphological defects	↓ (−20%)	ND	NS	↑ (−67%)	↓ (−70%)
		Number/concentration	↑ (+6%)	↓ (−30%)	NS	↓ (−20%)	↑ (+60%)
		Motility	ND	↓ (−30%)	NS	↓ (−20%)	↑ (+40%)
		Viability	ND	NS	ND	ND	ND
References			(65)	(129)	(63)	(63)	(63)
**Species**			**Rat**	**Rabbit**	**Rabbit**	**Fish**	
Pathology			Obesity (HFD)	Control	Diabetic (alloxan)	Control	
Metformin treatment			Yes	Yes	Yes	Yes	
Comparison type			Comparison HFD with HFD + metformin	Comparison untreated with metformin	Comparison untreated diabetic with metformin diabetic	Comparison untreated with metformin	
Administration mode			Gavage 100 mg/kg during 8 weeks	Gavage 120 mg/kg/day during 3 months	Gavage 120 mg/kg/day during 3 months	Water with 40 μg/L metformin (exposition: 3 days after hatching and for 365 days)	
Sperm	*in vivo*	Morphological defects	↓ (+7%)	↑ (+44%)	↑ (+60%)	Low sperm production due to intersex gonad (partly convert in ovary with oocytes)	
		Number/concentration	↑ (+10%)	↓ (−30%)	↓ (−87%)	↓ clutch size (−80%)	
		Motility	↑ (+20%)	↓ (−12%)	↓ (−40%)	ND	
		Viability	↑ (+20%)	↑ (+85% dead)	NS	ND	
References			(66)	(71)	(71)	(112)	

*NS, not significant; HFD, high fat diet; ND, not determined*.

Other studies have exposed rodents (rat, rabbit) to metformin in different metabolic models. Either diabetes or obesity induced by fatty acid-rich diet have shown that metformin could limit the decrease in testicular weight, and the thickness of the seminiferous epithelium (66, 69, 70). Depending on the report, the testosterone concentrations and sperm concentrations were improved following the treatment (63, 65, 66, 71). In rabbit, the metformin treatment showed a negative effect on concentration, mobility and number of morphological abnormalities of spermatozoa (71).

In birds, metformin (1 mM) treatment increased viability, mobility and acrosomal response of chicken sperm (72). During chicken seminiferous tubule culture, 48 h of metformin treatment (5 mM) induced a decrease in the rate of proliferative germ cells number (73). This effect was opposite to that observed in equines. Metformin (up to 10 mM) did not induce any effects on viability and mobility of sperm or phosphorylation of AMPK in horses (74).

In regards to the mechanism leading to decreased male fertility, and considering that in mice both the AMPK α1 and α2 subunits are expressed in Leydig, Sertoli and male germ cells (75) with a predominant expression of α1 subunit over α2 (75 vs. 25%) (76), it is possible that metformin impacts spermatogenesis and steroidogenesis in the testes partly through the AMPK pathway. It has been shown that AMPK activation by metformin in rat cultured Sertoli cells induces an increase in lactate production without changes in LDH (lactate dehydrogenase) activity. However, a decrease in the expression of MCT4 (monocarboxylate transporter 4), GLUT1, GLUT3 and an increase in the concentrations of alanine and acetate were also reported (77) suggesting activation of glycolysis without concomitant increase in mitochondrial bioenergetics.

Collectively, these studies suggested that obesity (or the metabolic changes associated with this condition) or a high fat diet may set the basis of an increased susceptibility to infertility issues. Metformin (in a dose, biological sample, and species-specific manner) treatment has the potential to activate targets resulting in an overall improvement of fertility.

## Metformin and steroidogenesis

In females, the impact of metformin on androgen synthesis is controversial (78–80). It is argued that metformin may reduce androgen levels indirectly through the resumption of ovulation. Several studies have shown that treatment with metformin induces a reduction in the hyperinsulinemia and hyperandrogenism that is associated with PCOS in obese and non-obese patients (81, 82). It has been suggested that metformin reduces hyperandrogenism through its ability to modulate both ovarian and adrenal androgen output, reducing LH secretion and increasing in some cases, sex hormone binding globulin. The ability of metformin to reduce the androgen levels seems to be variable according to the studies (83, 84).

Metformin has been shown to regulate steroidogenesis through a number of different mechanisms and cell types. Culture of luteinized granulosa cells exposed to the pre-ovulatory LH surge and with metformin lowers progesterone and estradiol syntheses in the same manner as non-luteinized granulosa cells exposed to either FSH or insulin (85). Rice et al. and Fuhrmeister et al. have demonstrated, that metformin induces a decrease in estradiol synthesis via the inhibition of aromatase expression by the MAPK signaling pathway (86, 87). However, metformin also induces activation of insulin-dependent AMPK pathways involved in lactate production by human granulosa cells (88, 89). Incubation of a human theca cell line with metformin induces a decline in androstenedione synthesis (90), possibly via the activation of AMPK.

In males, Tartarin et al. demonstrated that exposure of mice and human fetal testes to metformin decreases testosterone production. *In vivo*, administration of metformin resulted in a decrease in testosterone secretion, however, this effect was reversed when metformin administration was stopped (61). Conversely in humans, it appears that several months of treatment with metformin (850 mg/day during the first week, 1,700 mg/day during the second week, and 2,550 mg/day until the end of treatment period at 6 months) can increase the serum testosterone and LH levels of obese individuals.

*In vitro* studies have demonstrated that metformin significantly perturbs both androstenedione and testosterone syntheses in theca cells (90). Incubation of primary cultures of rat Leydig cells in the presence of an activator of AMPK, resveratrol, decreases hCG-induced testosterone synthesis by inhibition of P450c17 and StAR (91). Moreover, it have been demonstrated that metformin decreases the capacity of Leydig cells to secrete progesterone (92). In rats and cows, the incubation of granulosa cells with metformin also induces a decline in steroid synthesis that is correlated with an increase in AMPK phosphorylation. It appears that progesterone synthesis falls in the presence of metformin alone, but also during stimulation with FSH, IGF-1, or both (93, 94). This decrease can be explained by a decrease in the expression of some steroidogenic enzymes (3-β HSD in the rat and 3-β HSD, CYP11A1, and StAR in the cow), an effect which is not observed in rats (95).

In human primary breast adipose tissue, consequences of metformin exposure revealed a significant decrease in the forskolin/phorbol ester induced aromatase expression (96).

Thus, based on the available literature, metformin acting via a number of mechanisms has the ability to modulate steroid levels both *in vivo* and *in vitro*.

## *In utero* exposure and gonadal development

Metformin is the treatment of choice in cases of pregnancy disorders, such as gestational diabetes mellitus or preeclampsia (97). One hypothesis is that metformin regulates preeclampsia via mitochondrial function especially in the placenta and expression of antiangiogenic factors (97). However, the maternal administration of metformin reaches the fetus with umbilical cord concentrations (on average 457 ± 335 μg/L, equivalent to 3 μM) similar to those found in the maternal circulation (730 ± 440 μg/L, equivalent to 5 μM) (98, 99). Salomaki et al. reported serum concentrations of only 0.174 and 0.130 μM in the mother and fetus, respectively, at 24 h post-administration of daily doses of 300 mg/kg throughout pregnancy (100), suggesting that metformin may accumulate in certain tissues at higher concentrations than in plasma (101).

Considering that there are no reports of metformin inducing teratogenicity, the long-term health consequences of *in utero* metformin exposure remains elusive, primarily due to limited study designs (102, 103). *In utero* metformin-exposure resulted in children that were heavier, and with larger head size at 18 m of age (104). Recently, Hanem et al. demonstrated that *in utero* exposure to metformin resulted in children with a higher body mass index (BMI) and increased prevalence of overweight/obesity at 4 years of age compared to children of the placebo group (105), indicating that in humans, metformin administered during pregnancy has the ability to alter anthropometrics in the offspring.

But what is known on the effects of metformin on gonadal development during *in utero* exposure? Very limited studies exist, focusing mainly on the effects of metformin on male offspring. From these, no effect on testicular size was reported for young boys between 2.5 and 7 years of age born to mothers affected with gestational diabetes treated with either insulin or metformin and insulin (106). However, testicular size was not compared to boys born from placebo mothers and to individuals that had not yet reached the age of puberty. Tartarin et al. observed that embryonic exposure of mice to metformin during the first half of pregnancy had a negative impact on the testicular size of young mice and number of Sertoli cells (at 16.5 dpc and 1 dpp) (61). At 16.5 dpc, a decrease in testicular testosterone concentration and Leydig cell count was also observed but was no longer found at birth. This suggests that while metformin is able to modulate mammalian testis development, some plasticity in the ability for the testis to recover exists during perinatal periods. Nonetheless, the long-term effects on fertility are yet to be determined.

In females with early symptoms of precocious puberty associated with hyperinsulinemia, metformin administration has been shown to delay the onset of clinical puberty and the pubertal increase in IGF1 levels (107, 108). Moreover, there was also a metformin-associated delay of menarche (108). The mode of action whereby metformin is able to delay pubertal onset and progression in girls remains to be understood. The observed delay of menarche appeared to be associated with falls in adiposity and insulin, leptin and IGF1 concentrations (107), suggesting that the effects of metformin on the ovary seemed to be indirect.

While exposure to metformin is usually through therapeutical administration, in the last decade, due to the increased use of metformin, accumulation of this drug has been reported in wastewater, drinking water and cosmetics, making it one of the 14 most active pharmacological molecules in the environment (109–111) with concentration reaching between 10 and 100 μg/L (1 μM) (112). Thus, it is possible that ingestion of metformin-containing water and/or use of metformin-containing cosmetics may elicit unwanted effects on humans as well as aquatic species exposed to metformin. Indeed, a 360-days long exposure of male fish (Pimephales promelas) to metformin (40 μg/l) leads to the appearance of an “intersex” gonads (113), with no intersex phenotype observed in mammals (61, 106). Gonadal estrogen and aromatase function play an important role in the gonad determinism in fish and avian species, thus, disruption of steroid production could lead to modification on gonadal development in these species. However, the exposure of cyp19a1b-GFP zebrafish model (GFP-driven promoter of aromatase in the central nervous system) to metformin (0.3–30 μM) or its derivative guanylurea (0.08–7 μM) did not result in changes in GFP expression suggesting a specific regulation of different aromatase transcripts by metformin depending on the promoter and tissue (gonad vs. central nervous system).

## Metformin and epigenetics

*In utero* metformin exposure has been described safe for the fetus. Some evidence suggests that the beneficial effects of metformin are partly AMPK-dependent to counteract stress (114). However, it is important to follow the development of the offspring until adulthood to evaluate the risk to develop metabolic disorders through epigenetic information (114). Thus, if metformin is provided with a high-fat diet, then a significant increase in visceral fat depot of the offspring is observed during adulthood (99). In rodents, prenatal exposure to metformin modifies the hepatic fetal imprinting resulting in changes in the expression of several genes involved in the metabolism of cholesterol, lipids, fatty acids and steroids. Moreover, it decreases the expression of insulin-sensitive glucose transporter, GLUT4, in epididymal adipose tissue suggesting long-term effects, such as glucose intolerance in the testis (99). In a follow-up study of metformin in a gestational diabetes trial showed that prenatal exposure had a change in the pattern of fat distribution in children at 2 years-old (same body fat mass but more subcutaneous fat (102). Taken together these studies point to a putative epigenetic effect of metformin which could be exerted during perinatal periods.

As possible mechanisms involved in epigenetics, indicated the section of Cellular Targets of Metformin, metformin directly targets the H3K27me3 demethylase KDM6A/UTX resulting in global augmentation of H3K27me3 levels in cultured cells *in vitro* and *in vivo* (23). Moreover, several studies reported DNA hypermethylation following metformin treatment via its effect on one-carbon metabolism (115, 116). This increase in DNA methylation is probably due to an increase in the activity of *S*-adenosylhomocysteine hydrolase (SAHH). This enzyme hydrolyzes *S*-adenosylhomocysteine (SAH), a strong feedback inhibitor of *S*-adenosyl-L-methionine-dependent methyltransferases including DNA methyltransferases (DNMTs). Treating endometrial or ovarian cancer cells with metformin results in a decrease of the histone H19 levels and enables DNMT3B to increase DNA methylation (115). However, no statistically significant effect of metformin was observed in plasma homocysteine (metabolite that plays a critical role in DNA methylation) concentrations in PCOS patients with or without metformin treatment (117). A subgroup analyses suggested that metformin might induce Hcy accumulation when administered without folic acid or B-group vitamins supplementation (117). Further studies are warranted at demonstrating the links amongst metformin, B-group vitamins, and DNA methylation in patients with PCOS or infertility.

Alternatively, metformin could regulate epigenetic reprogramming through the activation of AMPK. A recent review reported the different mechanisms involved in the histone modifications in response to metformin-induced activation of AMPK phosphorylation of HATs (histone acetyltransferases), increased SIRT1 activity, and inhibition of class II HDACs (histone deacetylases) (118–120). AMPK has been shown to phosphorylate histone H2B by regulating HDAC in mouse embryonic fibroblasts (121). Metformin was shown to inhibit ovarian cancer via decreasing H3K27 trimethylation in an AMPK-dependent manner (122). Studies on Sertoli cells lacking AMPKα1 have highlighted the role of the α1 subunit of AMPK (123) metabolic activity and the secretion of many metabolites, such as glycine, malonate, succinate and alanine, which may act on the enzymes modifying epigenetic marks. Oocytes lacking α1AMPK, a hyperacetylation of histone H3 and a decrease in the activity of SIRT1 is detected (123). Interestingly, the effects of metformin on HDACs are dissimilar because it increases the expression and/or activity of the class III HDAC SIRT1 (124–127) and pharmacological doses of metformin in the cryopreservation media of mouse sperm in2duced SIRT1 activity (128).

Taken together, while these studies show a link between metformin and DNA methylation status, many precise aspects of this link still need to be clarified.

## Conclusion

After half a century, metformin has established itself as a first defense against insulin-dependent morbidities and undoubtedly has become a useful drug for improving fertility outcomes in both male and female patients. Metformin can modify testis and ovary function directly through AMPK-dependent and independent mechanisms. Its effects include improved sperm function and fertilization rates, oocyte quality and embryo development and reduction in miscarriage rates. The general consensus in the literature is that metformin is considered safe to use during pregnancy in regards to perinatal outcomes. However, adverse effects of metformin in the germ cell populations of offsprings exposed *in utero* and those on subsequent generations are less clear. While our understanding of the effects of metformin is continually progressing, further research is needed to have a more complete understanding of metformin's impact on fertility.

## Author contributions

MF, MB, RK, AB, FB, CG, JD, and PF: redaction of different parts of the review.

### Conflict of interest statement

The authors declare that the research was conducted in the absence of any commercial or financial relationships that could be construed as a potential conflict of interest.
